# Prognostic value of combined preoperative fibrinogen and neutrophil–lymphocyte ratio in patients with hepatocellular carcinoma after liver transplantation

**DOI:** 10.18632/oncotarget.13804

**Published:** 2016-12-07

**Authors:** Shun-Jun Fu, Fei Ji, Ming Han, Mao-Gen Chen, Xiao-Ping Wang, Wei-Qiang Ju, Qiang Zhao, Lin-Wei Wu, Qing-Qi Ren, Zhi-Yong Guo, Dong-Ping Wang, Xiao-Feng Zhu, Yi Ma, Xiao-Shun He

**Affiliations:** ^1^ Organ Transplant Center, the First Affiliated Hospital, Sun Yat-sen University, Guangzhou 510080, P. R. China; ^2^ Guangdong Provincial Key Laboratory of Organ Donation and Transplant Immunology, the First Affiliated Hospital, Sun Yat-sen University, Guangzhou 510080, P. R. China; ^3^ Guangdong Provincial International Cooperation Base of Science and Technology (Organ Transplantation), the First Affiliated Hospital, Sun Yat-sen University, Guangzhou 510080, P. R. China; ^4^ Department of Hepatobiliary Surgery, The Second Affiliated Hospital of Guangzhou University of Chinese Medicine, Guangdong Provincial Hospital of Traditional Chinese Medicine, Guangzhou 510120, China

**Keywords:** fibrinogen, neutrophil–lymphocyte ration, hepatocellular carcinoma, prognosis, liver transplantation

## Abstract

**Objectives:**

Elevated plasma fibrinogen (Fib) correlated with patient's prognosis in several solid tumors. However, few studies have illuminated the relationship between preoperative Fib and prognosis of HCC after liver transplantation. We aimed to clarify the prognostic value of Fib and whether the prognostic accuracy can be enhanced by the combination of Fib and neutrophil–lymphocyte ratio (NLR).

**Results:**

Fib was correlated with Child-pugh stage, alpha-fetoprotein (AFP), size of largest tumor, macro- and micro-vascular invasion. Univariate analysis showed preoperative Fib, AFP, NLR, size of largest tumor, tumor number, macro- and micro- vascular invasion were significantly associated with disease-free survival (DFS) and overall survival (OS) in HCC patients with liver transplantation. After multivariate analysis, only Fib and macro-vascular invasion were independently correlated with DFS and OS. Survival analysis showed that preoperative Fib > 2.345 g/L predicted poor prognosis of patients HCC after liver transplantation. Preoperative Fib showed prognostic value in various subgroups of HCC. Furthermore, the predictive range was expanded by the combination of Fib and NLR.

**Materials and Methods:**

Data were collected retrospectively from 130 HCC patients who underwent liver transplantation. Preoperative Fib, NLR and clinicopathologic variables were analyzed. The survival analysis was performed by the Kaplan-Meier method, and compared by the log-rank test. Univariate and multivariate analyses were performed to identify the prognostic factors for DFS and OS.

**Conclusions:**

Preoperative Fib is an independent effective predictor of prognosis for HCC patients, higher levels of Fib predict poorer outcomes and the combination of Fib and NLR enlarges the prognostic accuracy of testing.

## INTRODUCTION

Hepatocellular carcinoma (HCC) is an aggressive malignancy and the third most common cause of cancer-related deaths worldwide [1, 2]. The prevalence of cirrhosis among HCC patients was approximately 80% [3], making liver transplantation the optimal treatment. The golden criteria for HCC patient selection now is Milan criteria. However, though excellent outcomes in patients meeting the Milan criteria, nearly 15% to 20% of patients still develop tumor recurrence [4, 5]. Instead, some patients exceeding the Milan criteria may have favorable outcomes. For the Milan criteria is based only on preoperative imaging diagnosis, without consideration of the tumor biology. Moreover, biopsy is not very suitable for diagnosis and grading of HCC because of the risk of tumor seeding [6]. Therefore it is important to search for some effective preoperative serum biomarkers to identify patients at a high risk of recurrence or metastases, and provide personalized therapy to improve the clinical outcomes.

A rising evidence for an interactive relationship between the hematological system and tumor biology has been documented [7]. Tumor cells can activate the coagulation pathway, and the tumor-mediated activation has been demonstrated to promote angiogenesis, tumor growth and hematogenous metastasis [8, 9]. Fibrinogen (Fib) is a glycoprotein synthesized by hepatocytes. It plays an important role in coagulation and can be recognized by integrin and nonintegrin receptors on various types of cells, such as, stromal, inflammatory and tumorous cells. These fibrinogen-mediated receptors have been reported to control cell proliferation, apoptosis, and migration as well as the expression of inflammatory mediators [10–12]. Davalos [13] had also revealed Fib played a proinflammatory role in cancer. Another proinflammatory factor affecting the prognosis is neutrophil–lymphocyte ratio (NLR) index. It reflects inflammatory response of the host to cancer as well as the systemic inflammation mediated by tumor [14]. Previous studies have shown that preoperative hyperfibrinogenemia and NLR were associated with the progression and poor prognosis of several cancers, such as colorectal [15], gastric [16], pancreatic [17], and lung cancer [18], biliary tract cancer [19], HCC [20]. However, the relationship between plasma Fib concentration and prognosis of HCC patients with after liver transplantation is not very clear, especially the prognostic value of Fib in combination with NLR has not been determined.

Therefore, this study aimed to clarify the prognostic value of Fib in HCC after liver transplantation, and to demonstrate whether the prognostic accuracy can be enhanced by the combination of Fib and NLR.

## RESULTS

### Clinicopathologic characteristics of patients

The study included 121 male patients (93.1%) and 9 female patients (6.9%), with a median age of 49.5 years (range 13–72 years). Of 130 patients, 119 (91.5%) were infected by hepatitis B virus (HBV). The Milan criteria [22], the University of California at San Francisco (UCSF) criteria [23], and the Hangzhou criteria were used as classification standards [24]. Of the 130 patients, 46 (35.4%) met the Milan criteria, 69 (53.1 %) met the UCSF criteria, and 81 (62.3 %) met the Hangzhou criteria.

### Determination of cut-off value

**Figure 1:**
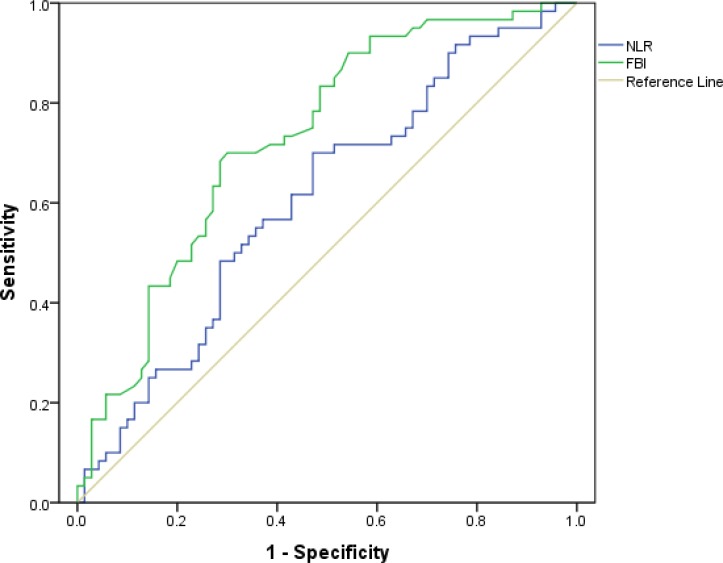
Determination of the cut-off value for Fib, NLR in HCC patients with liver transplantation

The cut-off values of preoperative Fib and NLR were determined using receiver operating characteristic (ROC) curve. The optimal cut-off value of Fib was 2.345 g/L, with a sensitivity of 70% and a specificity of 70% (the area under ROC curve: 0.729, 95% CI: 0.643–0.816, *P* < 0.001). The optimal cut-off value of NLR was 1.84, with a sensitivity of 70% and a specificity of 52.9% (the area under ROC curve: 0.608, 95% CI 0.511–0.705, *P* = 0.034) (Figure 1).

### Associations of Fib and NLR with clinicopathologic characteristics of HCC

The associations of preoperative Fib and NLR with clinicopathologic variables of patients with HCC were investigated, and the data showed that preoperative Fib was correlated with Child-pugh stage (*P* < 0.001), alpha-fetoprotein (AFP) (*P* = 0.005), size of largest tumor (*P* < 0.001), macro-vascular invasion (*P* = 0.003), and micro-vascular invasion (*P* < 0.001). Similarly, NLR was correlated with AFP (*P* = 0.020). However, there were no associations between preoperative Fib/NLR and other clinicopathologic features such as age, gender, HBsAg, preoperative therapy, tumor number, Edmonson grade. No significance was also found between NLR and size of largest tumor, macro-vascular invasion, micro-vascular invasion (all *P* > 0.05, Table 1).

**Table 1:** Relationship between preoperative plasma fibrinogen levels and clinicopathological characteristics

Category	Subcategory	Cases	Fib (g/L)		*P* value	NLR		*P* value
			≤ 2.345	> 2.345		≤ 1.84	> 1.84	
Gender	Male	121	60(49.6%)	61(50.4%)		50(41.3%)	71(58.7%)	
	Female	9	7(77.8%)	2(22.2%)	0.103	5(55.6%)	4(44.4%)	0.404
Age (years)	≤ 50	68	30(44.1%)	38(55.9%)		26(38.2%)	42(61.8%)	
	< 50	62	37(59.7%)	25(40.3%)	0.076	29(46.8%)	33(53.2%)	0.325
HBsAg	Positive	119	61(51.3%)	58(48.7%)		51(42.9%)	68(57.1%)	
	Negative	11	6(54.5%)	5(45.4%)	0.835	4(36.4%)	7(63.6%)	0.677
Child- pugh stage	A	84	33(39.3%)	51(60.7%)		38(45.2%)	46(54.8%)	
	B	36	25(69.4%)	11(30.6%)		12(33.3%)	24(66.7%)	
	C	10	9(90.0%)	1(10.0%)	< 0.001	5(50.0%)	5(50.0%)	0.422
Preoperative tumor therapy	Yes	59	27(45.8%)	32(54.2%)		28(47.5%)	31(52.5%)	
	No	71	40(56.3%)	31(43.7%)	0.230	27(38.0%)	44(62.0%)	0.085
AFP(ng/ml)	≤ 400	82	50(61.0%)	32(39.0%)		41(50.0%)	41(50.0%)	
	> 400	48	17(35.4%)	31(64.6%)	0.005	14(29.2%)	34(70.8%)	0.020
Size of largest	≤ 5	74	51(68.9%)	23(31.1%)		38(51.4%)	36(48.6%)	
	5 to 8	22	11(50.0%)	11(50.0%)		7(31.8%)	15(68.2%)	
tumor (cm)	> 8	34	5(14.7%)	29(85.3%)	< 0.001	10(29.4%)	24(70.6%)	0.055
Tumor Number	≤ 3	93	51(54.8%)	42(45.2%)		40(43.0%)	53(57.0%)	
	> 3	37	15(40.5%)	22(59.5%)	0.141	15(40.5%)	22(59.5%)	0.797
Edmonson	I-II	86	45(52.3%)	41(47.7%)		38(44.2%)	48(55.8%)	
Grading	III-IV	44	22(50.0%)	22(50.0%)	0.802	17(38.6%)	27(61.4%)	0.544
Macro-vascular invasion	Yes	29	8(27.6%)	21(72.4%)		10(34.5%)	19(65.5%)	
	No	101	59(58.4%)	42(41.6%)	0.003	45(44.6%)	56(55.4%)	0.333
Micro-vascular invasion	Yes	20	2(10.0%)	18(90.0%)		5(25.0%)	15(75.0%)	
	No	110	65(59.1%)	45(40.9%)	< 0.001	50(45.5%)	60(54.5%)	0.089
Milan	Within	46	35(76.1%)	11(23.9%)		23(50.0%)	23(50.0%)	
criteria	Beyond	84	32(38.1%)	52(61.9%)	< 0.001	32(38.1%)	52(61.9%)	0.189
UCSF	Within	69	48(69.6%)	21(30.4%)		34(49.3%)	35(50.7%)	
criteria	Beyond	61	19(45.2%)	42(54.8%)	< 0.001	21(34.4%)	40(65.6%)	0.087
Hangzhou	Within	81	56(69.1%)	35(30.9%)		39(48.1%)	42(51.9%)	
criteria	Beyond	49	11(22.4%)	28(77.6%)	< 0.001	16(32.7%)	33(67.3%)	0.083

Fib: Fibrinogen; HBsAg: Hepatitis B surface antigen; AFP: Alpha fetoprotein.

### Independent prognostic factors for HCC patients after liver transplantation

To further identify predictors of postoperative DFS and OS, Fib and the clinicopathologic parameters were evaluated using univariate and multivariate analyses. The results revealed that preoperative Fib (*P* < 0.001), Child-Pugh stage (*P* = 0.037), AFP (*P* < 0.001), size of largest tumor (*P* < 0.001), tumor number (*P* < 0.001), macro-vascular invasion (*P* < 0.001), micro-vascular invasion (*P* < 0.001) and NLR (*P* = 0.013) were responsible for the DFS. Similarly, significant factors of OS included Fib (*P* < 0.001), AFP (*P* < 0.001), size of largest tumor (*P* < 0.001), tumor number (*P* < 0.001), macro-vascular invasion (*P* < 0.001), micro-vascular invasion (*P* < 0.001), NLR (*P* = 0.041) (Table 2). The multivariate model analysis showed that Fib, AFP, macro-vascular invasion, tumor number were independent predictors of DFS (all *P* < 0.05), whereas Fib, size of largest tumor, macro-vascular invasion were independent predictors of OS (all *P* < 0.05) (Table 3).

**Table 2:** Influence of clinicopathological characteristics on patients’ prognosis

Variables	*n*	DFS				OS			
		1-yr	3-yr	5-yr	*P*	1-yr	3-yr	5-yrs	*P*
Gender									
Male	121	71.1%	56.0%	47.2%		85.1%	63.4%	56.5%	
Female	9	77.8%	66.7%	66.7%	0.382	88.9%	77.8%	77.8%	0.539
Age (years)									
≤ 50	68	64.7%	52.1%	42.6%		80.9%	57.7%	49.0%	
> 50	62	79.0%	61.8%	54.9%	0.152	90.3%	71.7%	67.2%	0.060
HBsAg									
Positive	119	72.3%	57.9%	49.1%		85.7%	65.6%	58.5%	
Negative	11	63.6%	42.4%	42.4%	0.402	81.8%	63.6%	54.5%	0.780
Child-Pugh stage									
A	84	66.7%	48.7%	39.3%		83.3%	60.9%	56.0%	
B	36	80.6%	69.0%	63.3%		74.1%	67.7%	54.4%	
C	10	80.0%	80.0%	80.0%	0.037	80.0%	80.0%	80.0%	0.364
Preoperative tumor therapy									
Yes	59	72.9%	53.1%	50.7%		86.4%	66.3%	52.6%	
No	71	70.4%	59.8%	52.0%	0.580	84.5%	62.8%	60.9%	0.960
AFP (ng/ml)									
≤ 400	82	84.1%	72.5%	60.7%		92.7%	77.5%	70.8%	
> 400	48	75.0%	29.6%	26.3%	< 0.001	72.9%	41.7%	36.4%	< 0.001
Size of largest tumor (cm)									
≤ 5	74	86.5%	75.3%	71.2%		95.9%	84.2%	78.6%	
5 to 8	22	68.2%	38.4%	25.6%		77.3%	54.2%	36.9%	
> 8	34	41.2%	26.7%	16.7%	< 0.001	67.6%	29.4%	29.4%	< 0.001
Tumor number									
≤ 3	93	81.7%	69.1%	59.8%		87.1%	77.1%	68.3%	
> 3	37	45.9%	25.3%	20.2%	< 0.001	81.1%	31.3%	31.3%	< 0.001
Edmondson grading									
I–II	86	73.3%	55.1%	46.4%		87.2%	65.9%	55.7%	
III–IV	44	68.2%	59.1%	51.7%	0.812	81.8%	61.1%	61.1%	0.799
Macro-vascular invasion									
Yes	29	44.8%	8.0%	4.0%		72.4%	23.6%	19.7%	
No	101	79.2%	70.7%	61.7%	< 0.001	89.1%	76.7%	69.6%	< 0.001
Micro-vascular invasion									
Yes	20	50.0%	20.8%	13.9%		75.0%	27.3%	21.8%	
No	110	75.5%	63.0%	54.7%	< 0.001	87.3%	71.1%	64.7%	< 0.001
Fib (g/L)									
≤ 2.345	67	88.1%	75.6%	71.0%		95.5%	84.6%	77.1%	
> 2.345	63	54.0%	36.1%	25.7%	< 0.001	74.6%	42.5%	37.3%	< 0.001
NLR									
≤ 1.84	55	80.0%	68.0%	65.2%		90.9%	74.2%	66.8%	
> 1.84	75	65.3%	48.5%	39.4%	0.013	81.3%	56.7%	51.0%	0.041
Milan criteria									
Within	46	93.5%	88.7%	85.3%		97.8%	95.6%	89.9%	
Beyond	84	59.5%	38.5%	28.5%	< 0.001	78.6%	47.3%	40.8%	< 0.001
UCSF criteria									
Within	69	91.3%	80.4%	78.1%		94.2%	89.5%	81.9%	
Beyond	61	49.2%	29.3%	15.9%	< 0.001	75.4%	35.8%	31.3%	< 0.001
Hangzhou criteria									
Within	81	87.7%	80.9%	71.8%		92.6%	84.8%	79.9%	
Beyond	49	44.9%	16.3%	10.2%	< 0.001	73.5%	31.4%	24.2%	< 0.001

DFS: disease-free survival; OS: overall survival. Other abbreviations as in Table 1.

**Table 3:** Prognostic factors for DFS and OS by multivariate cox proportional hazards regression model

Variables	DFS			OS		
	HR	95%CI	*P*	HR	95%CI	*P*
Macro-vascular invasion	2.240	1.199–4.183	0.011	2.430	1.302–4.537	0.005
Size of largest tumor				1.562	1.066–2.289	0.022
Tumor number	2.112	1.169–3.815	0.013			
AFP	2.018	1.166–3.491	0.012			
Fib	2.582	1.442–4.626	0.001	2.015	0.998–4.069	0.051

HR: hazard ratio; CI: confidence interval. Other abbreviations as in Table 2 and 3.

### Association of Fib with overall and disease-free survival rates

**Figure 2:**
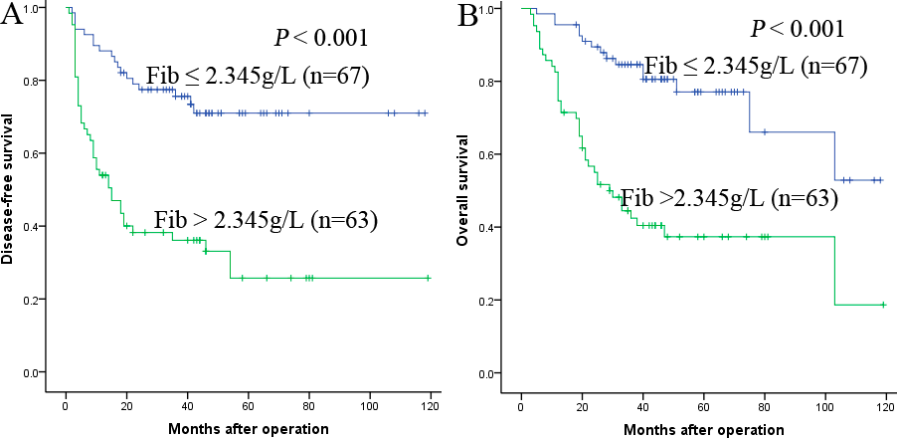
Relationship between Fib and DFS/OS of HCC patients after liver transplantation (**A**) DFS of patients with Fib > 2.345 g/L was significantly shorter than those with Fib ≤ 2.345 g/L (*P* < 0.001, log-rank test). (**B**) OS of patients with Fib > 2.345 g/L was also markedly shorter than those with Fib ≤ 2.345 g/L (*P* < 0.001, log-rank test).

To determine the prognostic value of Fib in predicting OS and DFS, the 130 HCC patients were divided into two groups: the Fib ≤ 2.345 g/L group (*n*= 67) and the Fib > 2.345 g/L group (*n* = 63). The patients’ survival was analyzed using the Kaplan-Meier method. The data showed that the 1-, 3- and 5-year DFS rates were significantly higher in the Fib ≤ 2.345 g/L group than in the Fib > 2.345 g/L group (88.1%,75.6%, and 71.0% vs 54.0%, 36.1% and 25.7%, respectively, *P* < 0.001) (Figure 2A), and the 1-, 3- and 5-year OS rates were also markedly higher in the Fib ≤ 2.345 g/L group than in the Fib > 2.345 g/L group (95.5%, 84.6% and 77.1% vs 74.6%, 42.5% and 37.3%, respectively, *P* < 0.001) (Figure 2B). Therefore, our research suggested that the elevation of preoperative Fib were correlated with a poor survival.

### Prognostic values of preoperative Fib in different HCC subgroups

**Figure 3:**
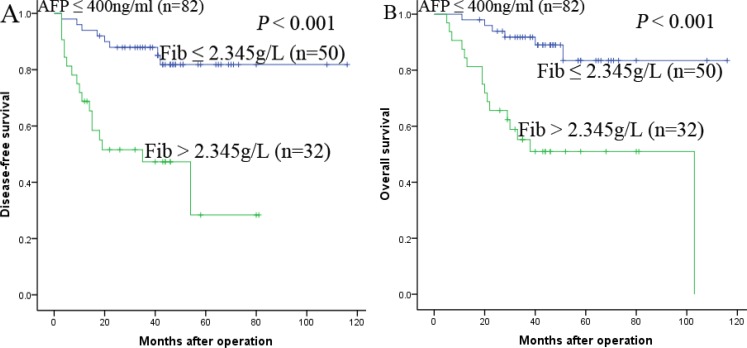
Kaplan-Meier survival curves of patients with AFP ≤ 400ng/ml subgroup Fib > 2.345 g/L significantly correlated with shorter DFS (**A**) and OS (**B**).

**Figure 4:**
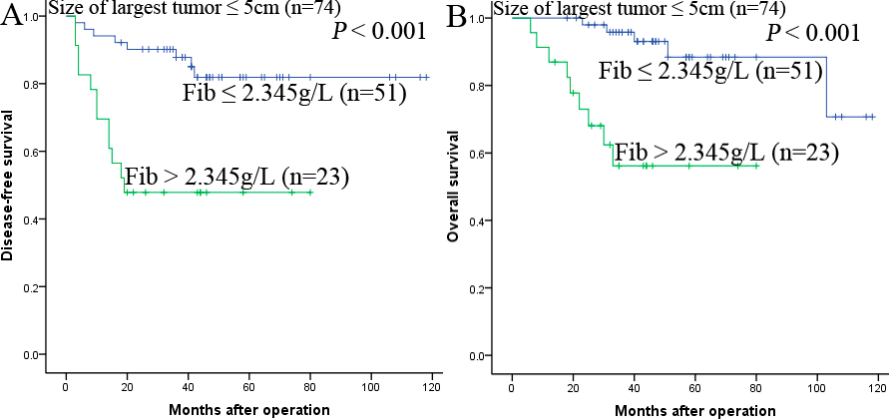
Kaplan-Meier survival curves of patients with size of largest tumor < 5 cm subgroup Fib > 2.345 g/L significantly correlated with shorter DFS (**A**) and OS (**B**).

**Figure 5:**
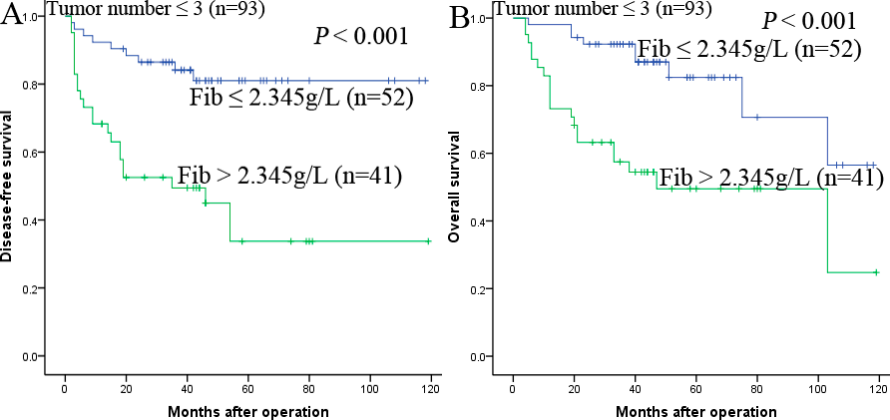
Kaplan-Meier survival curves of patients with tumor number ≤ 3 subgroup Fib > 2.345 g/L significantly correlated with shorter DFS (**A**) and OS (**B**).

**Figure 6:**
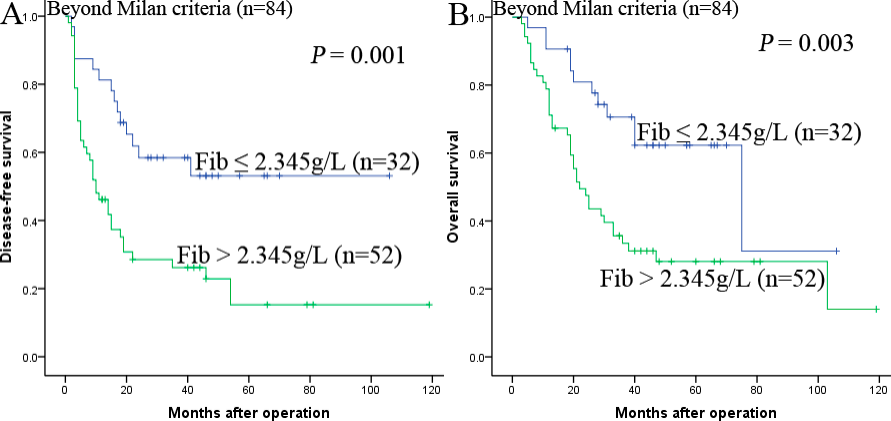
Kaplan-Meier survival curves of patients beyond Milan criteria Fib > 2.345 g/L significantly correlated with shorter DFS (**A**) and OS (**B**).

The research above proved that preoperative Fib was an independent prognostic factor and significantly correlated with DFS and OS. We further analyzed the prognostic ability of preoperative Fib in different subgroups of HCC patients. The results displayed that Fib was a prognostic indicator for DFS (68.8%, 47.3%, 28.4% vs 94.0%, 87.9%, 81.8%, *P* < 0.001, respectively) and OS (84.4%, 55.2%, 51.0% vs 98.0%, 91.8%, 83.4%, *P* < 0.001, respectively) in patients with AFP ≤ 400ng/ml (Figure 3A, 3B). In addition, in the subgroup of the largest tumor size ≤ 5 cm, Fib also appeared noticeable prognostic value in predicting poorer DFS (69.6%, 47.8%, 47.8%. vs 94.1%, 87.8%, 81.9%, *P* < 0.001, respectively) and OS (87.0%, 56.2%, 56.2% vs 100.0%, 95.8%, 88.4%, *P* < 0.001, respectively) (Figure 4A, 4B), and this prognostic ability of DFS (68.3%, 49.4%, 33.7% vs 92.3%, 84.1%, 81.0%, *P* < 0.001, respectively) and OS (73.2%, 57.5%, 49.5% vs 98.1%, 92.3%, 82.4%, *P* < 0.001, respectively) also existed in patients with tumor number ≤ 3 (Figure 5A,5B) and in patients with beyond Milan criteria (Figure 6A,6B) ( DFS: 46.2%, 26.2%, 15.3% vs 81.3%, 58.4%, 53.1%, *P* = 0.001, respectively; OS: 71.2%, 33.4%, 28.1% vs 90.6%, 70.6%, 62.3%, *P* = 0.003, respectively). These results further demonstrated that Fib had a more powerful prognostic ability than some other parameters on predicting the prognosis of HCC patients after liver transplantation, particularly in different kinds of HCC subgroups whose survival is so difficult to be predicted.

### Prognostic value of Fib in combination with NLR for HCC after liver transplantation

Fibrinogen had been demonstrated to play a proinflammatory role in cancer [13]. Qi [25] have shown that plasma fibrinogen levels are positively related with NLR. Hence, we decided to propose a novel prognostic marker based on a combined analysis of plasma fibrinogen and NLR. Patients were divided into three groups: group 1, NLR ≤ 1.84 and Fib ≤ 2.345 g/L; group 2, patients with NLR > 1.84 and Fib ≤ 2.345 g/L or with NLR ≤1.84 and Fib > 2.345 g/L; group 3, patients with both NLR > 1.84 and Fib > 2.345 g/L.

**Figure 7:**
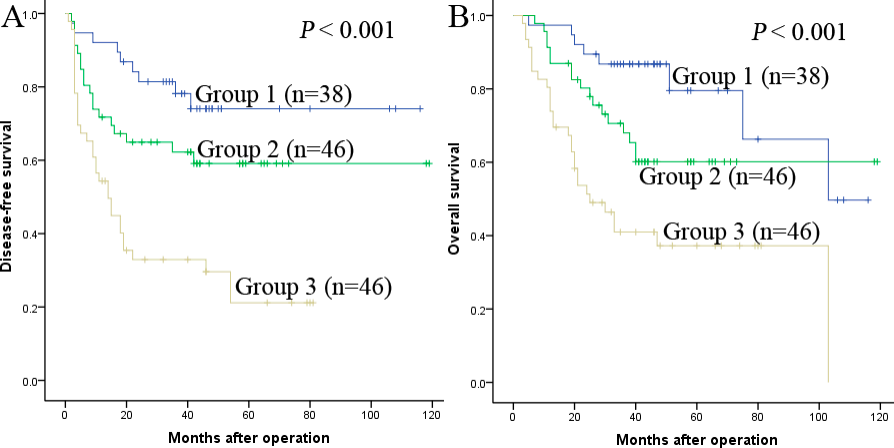
The combination of Fib and NLR was found to enhance prognostic accuracy for HCC Disease-free survival curves (**A**) and overall survival curves (**B**). Group1: both Fib ≤ 2.345 g/L and NLR ≤ 1.84; Group2: both Fib ≤ 2.345 g/L and NLR > 1.84 or both Fib > 2.345 g/L and NLR ≤ 1.84; Group3: both Fib > 2.345 g/L and NLR > 1.84.

The 1-, 3- and 5-year OS rates were markedly higher in group 1 than in group 2 and group 3 (97.4%, 86.8%, 79.5% vs 87.0%, 68.0%, 60.1% and 73.9% 41.0%, 37.2%, respectively, *P* < 0.001). Similarly, the 1-, 3- and 5-year DFS rates were also significantly higher in group 1 than in group 2 and group 3 (92.1%, 78.2%, 74.0% vs 71.7%, 62.2%, 59.1% and 54.3%, 32.9%, 21.2%, respectively, *P* < 0.001) (Figure 7A and 7B). Furthermore, we found that the 1-, 3- and 5-year DFS and OS rates were both significantly higher in group 2 than in group 3 (*P* = 0.002 and *P* = 0.008) ( Figure 7A and 7B).

## DISCUSSION

More and more studies have verified that the haemostatic system played an important role in cancer [25, 26]. The haemostatic system can maintain the vascular integrity and also seems to play role in vascular development, cell adhesion, tissue repair, and transendothelial cell migration. Therefore, haemostatic system components are prior to be tumor progression factor candidates [27].

Fibrinogen, a dimeric glycoprotein, is synthesized by hepatocytes. As a key substance in clot formation, fibrinogen contributes in wound healing. After being converted to fibrin, it accelerates platelet aggregation by binding to platelets. Several studies have shown that many types of malignant cells overexpress fibrinogen receptors [28, 29]. So the fibrinogen may take part in the adhesive interaction among tumor cells or endothelial cells, and lead to hematogenous metastasis [30, 31]. Elevated levels of plasma fibrinogen are reported to be an useful prognostic predictor for several types of human cancers [15–19]. So we aim to clarify the prognostic ability of fibrinogen in HCC patients after liver transplantation.

In our study, we identified the cut-off value of preoperative Fib according to the ROC curve at first, 2.345 g/L was showed to be the optimal cut-off value with a maximum joint sensitivity and specificity. Interestingly, concerning the correlation between preoperative Fib and clinical characteristics, we found that an elevated Fib was positively related to Child-pugh stage, AFP, size of largest tumor, macro- and micro-vascular invasion. So these data indicated that the preoperative Fib could reflect the tumor burden and tumor progression. By univariate analysis, we found many significant prognostic predictors for DFS or OS of HCC, including preoperative Fib, Child-Pugh stage, AFP, size of largest tumor, tumor number, macro- and micro-vascular invasion. After multivariate analysis, however, we discovered that only preoperative Fib and macro-vascular invasion were significant independent prognostic factors of DFS and OS.

Through a further analysis, we caught that a shorter survival of HCC patients after LT with preoperative Fib > 2.345 g/L had been recorded in this study. The 1-, 3- and 5-year DFS and OS rates of patients with high level of preoperative Fib were markedly poorer than the low level group. The mechanisms may be that fibrinogen fragments can promote neovascularization and cell adhesion in tumor [32–33], whereas fibrinogen depositing in tumor tissue facilitates tumor metastasis by serving as an extracellular matrix for the adhesion and migration of tumor cells [34–35]. So preoperative fibrinogen levels may be used to predict tumor progression and metastasis.

In order to elucidate the prognostic ability of Fib in different subgroups of HCC patients, we found that Fib had noteworthy prognostic value for both DFS and OS in patients AFP ≤ 400 ng/ml, namely, the preoperative Fib can predict the prognosis for patients whose AFP is negative. In addition, in the subgroup of the largest tumor size ≤ 5 cm, tumor number ≤ 3 and beyond milan criteria, preoperative Fib > 2.345 g/L also appeared noticeable prognostic value in predicting poorer DFS and OS. Therefore, these results provided adequate evidence that preoperative Fib could act as a potential prognostic marker to predict survival in HCC patients after liver transplantation, particularly for the different kinds of HCC subgroups.

Both Fib and NLR are proinflammatary prognostic factors. Qi [25] had demonstrated a positive correlation betweem plasma fibrinogen levels and NLR. Arigami [36] had discussed the prognostic value of Fib in combination with NLR (F-NLR) in patients with esophageal squamous cell carcinoma, and found a close relationship between cancer progression and the F-NLR score. So in our study we tried to explore whether the prognostic value could be expanded by the combination of them. Noteworthiness, First, we found NLR was correlated with AFP. NLR was showed to be a significant prognostic factor for DFS/OS of HCC in univariate analysis, but not in multivariate model. Second, our research displayed that the combination of Fib and NLR predicted the prognosis better than either one alone, patients with NLR ≤ 1.84 and Fib ≤ 2.345 g/L had the highest DFS and OS rates, patients with NLR > 1.84 and Fib ≤ 2.345 g/L or with NLR ≤1.84 and Fib > 2.345 g/L were the second, then patients with both NLR > 1.84 and Fib > 2.345 g/L were the worst.

However, there are also some shortages in the present study. It is a retrospective and single-institution study. A well-designed, prospective study with larger number of patients is needed. Furthermore, owing to the relatively small sample of patients, we didn't divide the data into a training set and a test set for statistical validation.

In conclusion, our research verified that preoperative Fib could be a prognostic factor for predicting the prognosis of patients with HCC after liver transplantation, the combination of Fib and NLR enlarges the prognostic accuracy of testing. These findings may suggest us that when we make the treatment plan, we should also consider these prognosis-related serum biomarkers. Only in this way can we acquire better personalized treatment for HCC patients. In the future, the simple preoperative prognostic evaluation could be used to select patients for personalized therapy.

## MATERIALS AND METHODS

### Ethics statement

Written informed consent was provided to all patients prior to surgery. Study approval was granted by the independent ethics committees at the First Affiliated Hospital of Sun Yat-sen University. This study was conducted in accordance with the ethical standards of the World Medical Association Declaration of Helsinki.

### Study population

A total of 130 consecutive patients with hepatocellular carcinoma who had undergone liver transplantation from January 2008 to May 2013 were enrolled in this study. The patients were selected on condition of completed preoperative clinical, laboratory, imaging, and follow-up data. There were no relative drugs and interventions used which may directly influence the peripheral hematological components. Traditional methods for assessment of tumor extent, namely, preoperative magnetic resonance imaging (MRI) and computerized tomography (CT) were employed. Macroscopic vascular invasion was defined by gross involvement of the lobar or segmental branches of the portal or hepatic veins. Microscopic vascular invasion was defined by the presence of tumor emboli within the central hepatic vein, the portal, or the large capsular vessels [37, 38].

### Treatment and follow-up

All the operation were done in classic or modified piggyback fashion using well-described standard techniques [21]. All patients regularly received 20 mg basiliximab (Simulect, Novartis Pharma AG, Basel, Switzerland) during the operation and the fourth day after operation. FK506 and mycophenolate mofetil (MMF) were used to prevent rejection and maintain the immune suppression, the dose were determined by the reaction of patients. A routine concentration test of FK506 was carried out and immune suppressive drugs were adjusted based on the drug concentration and liver enzyme level, serum creatinine level and white blood cell count were also taken into consideration during the procedure. AFP and liver ultrasound were performed at each follow-up. Abdominal CT scan was performed every 6 months. Recurrence was defined as emergence of clinical, radiological, and/or pathological diagnosis of tumor as previously described. As for the patients with recurrence, the administration of sorafenib, TACE, RFA, tumor resection, and chemotherapy was selected due to specific conditions.

### Statistical analysis

Statistical analysis was performed using SPSS for Windows version 19.0 (SPSS, Chicago, IL, USA). Receiver operating characteristic (ROC) curve analysis was performed to select the most appropriate cut-off values for Fib and NLR. The χ2 test was used to compare categorical variables. The survival analysis was performed by the Kaplan-Meier method, and compared by the log-rank test. Univariate analysis of individual predictors of HCC recurrence or overall survival was performed using Kaplan-Meier method. Factors identified as significant (*P* < 0.1) on univariate analysis were entered into a multivariate competing risk Cox regression model to identify significant independent predictors of HCC recurrence and overall survival. The final multivariate model was performed using the forward stepwise procedure for variable selection. *P* < 0.05 was considered statistically significant.

## Abbreviations

HCC: hepatocellular cancer
NLR: neutrophil-lymphocyte ratio
Fib: fibrinogen
UCSF criteria: the University of California at San Francisco criteria
CT: computed tomography
MRI: magnetic resonance imaging
TACE: transcatheter arterial chemoembolization
PLT: platelet
AFP: alpha-fetoprotein
MMF: mycophenolate mofetil
ROC: receiver operating characteristics
DFS: disease-free survival
OS: overall survival
HBsAg: hepatitis B surface antigen
